# Complete Genome Sequences of Two *Helicobacter pylori* Strains from a Canadian Arctic Aboriginal Community

**DOI:** 10.1128/genomeA.00209-15

**Published:** 2015-04-16

**Authors:** Dangeruta Kersulyte, M. Teresita Bertoli, Sravya Tamma, Monika Keelan, Rachel Munday, Janis Geary, Sander Veldhuyzen van Zanten, Karen J. Goodman, Douglas E. Berg

**Affiliations:** a Department of Molecular Microbiology, Washington University Medical School, St. Louis, Missouri, USA; b Department of Medicine, Division of Infectious Disease, University of California San Diego, La Jolla, California, USA; c Department of Laboratory Medicine and Pathology, University of Alberta, Edmonton, Alberta, Canada; d Susie Husky Health Centre, Aklavik, Northwest Territories, Canada; e Department of Medicine, Division of Gastroenterology, University of Alberta, Edmonton, Alberta, Canada

## Abstract

We report here the complete genome sequences of two Amerind *Helicobacter pylori* strains from Aklavik, Northwest Territories, Canada. One strain contains extra iron-cofactored urease genes and ~140 rearrangements in its chromosome relative to other described strains (typically differing from one another by <10 rearrangements), suggesting that it represents a novel lineage of *H. pylori*.

## GENOME ANNOUNCEMENT

*Helicobacter pylori* is a genetically diverse gastric pathogen that chronically infects billions of people worldwide (1–3), including many residents of indigenous Arctic communities (4). Different sets of genotypes tend to predominate in different geographic regions or with different ethnic groups. Although strains from diverse peoples, including South American Amerindians, have been sequenced, no Arctic strains have been sequenced to date. Here, we report the complete genome sequences of two *H. pylori* strains from Aklavik, Northwest Territories, a Canadian Aboriginal community (~600 residents; 93% of Inuvialuit or Gwich’in ethnicity) with a high prevalence of severe *H. pylori*-associated gastritis (5–7).

Preliminary random amplified polymorphic DNA (RAPD) fingerprinting (8) placed 52 of 57 isolates into just six distinct groups, a homogeneity typical of isolated communities (9). Multilocus sequencing typing (MLST) (9) identified four RAPD groups and the five unique strains (43 isolates) as Amerind (more related to Amazonian than even East Asian strains). The other two RAPD groups exhibited European and hybrid Amerind/European MLSTs (4 and 10 isolates, respectively).

We completely sequenced (454 FLX with PCR-capillary to fill all gaps) (9, 10) two representative strains: Aklavik86, from the most abundant RAPD/MLST group (17 members), and Aklavik117, a unique RAPD/MLST Amerind type, distinguished by an *ftsZ*-linked IS*606* fragment PCR marker, which is common in Peruvian Amerind strains but rare in Aklavik (although present in Alaskan Native) strains and absent from non-Amerind *H. pylori* (9) (D. Kersulyte and D. E. Berg, unpublished data). Each strain contains a typical *H. pylori* chromosome size (1.5 kb or 1.6 kb) and two unrelated plasmids (<3 kb and 12.1 kb or 18.8 kb). BLASTn analyses of sequential 1-kb chromosomal segments confirmed that these two strains are of the Amerind type genome-wide.

PCR tests showed that Aklavik86 and Aklavik117 lack *cag* pathogenicity islands, as do all Aklavik Amerind strains, whereas most South American Amerind strains contain these islands (9). Aklavik117 does contain two TnPZ virulence-associated transposons, (11), one type I (fragmented) and one type II (intact), whereas Aklavik86 lacked TnPZs. Only two insertion sequence (IS) elements were present, IS*607* in Aklavik117 and IS*605* (deletion mutant) in Aklavik86, and no prophages were present in these two genomes.

Two Aklavik86 features merit special mention: (i) iron-dependent urease genes (previously observed only in helicobacters from carnivores [10, 12, 13]), next to *cheW*, as in *Helicobacter acinonychis* and *Helicobacter cetorum*, and (ii) an unprecedented ~140 rearrangements in its chromosome (identified using Mauve software) relative to Aklavik117 and all other sequenced *H. pylori* strains. This contrasts with ≤10 such rearrangements between most other *H. pylori* strain pairs (10, 14). Follow-up PCR tests for six Aklavik86-specific rearrangements identified each in most of the Aklavik Amerind strains. In contrast, no rearrangements were found in Aklavik117 or in one of the Amerind or one European RAPD group of Aklavik strains (11 and 10 isolates, respectively), or in any of 80 other strains variously from Peru, Europe, or Asia.

Aklavik86’s extraordinary iron-dependent urease genes and many chromosomal rearrangements suggest that it might represent a novel lineage, possibly derived from a jump many millennia ago from some ancient animal host. More generally, these findings should stimulate analyses of *H. pylori* population structure and evolution in human health and disease, especially in Arctic Aboriginal communities.

### Nucleotide sequence accession numbers. 

The complete *H. pylori* chromosome and plasmid sequences reported here have been deposited in GenBank under the accession numbers CP003476, CP003477, and CP003478 (Aklavik86) and CP003483, CP003484, and CP003485 (Aklavik117). The versions described in this paper are the first versions (CP003476.1, CP003477.1, and CP003478.1 [Aklavik86] and CP003483.1, CP003484.1, and CP003485.1 [Aklavik117]).
